# Cognitive Complaints in Schizophrenia: A Meta-Analysis of Studies Using the Subjective Scale to Investigate Cognition in Schizophrenia (SSTICS): Les plaintes cognitives dans la schizophrénie : Une méta-analyse des études utilisant la *Subjective Scale To Investigate Cognition in Schizophrenia* (SSTICS)

**DOI:** 10.1177/07067437261425086

**Published:** 2026-03-03

**Authors:** Naomi White, Stéphane Potvin, André Do, Emmanuel Stip

**Affiliations:** 1 5622 Centre de recherche de l’Institut Universitaire en Santé Mentale de Montréal, Montréal, Québec, Canada; 2 Department of Psychiatry and Addiction, 12368 Faculty of Medicine, University of Montreal, Montréal, Québec, Canada

**Keywords:** schizophrenia, cognition, insight, meta-analysis

## Abstract

**Background:** Subjective cognitive complaints, or neurocognitive insight, reflect to patients’ awareness of cognitive functioning. In schizophrenia, these complaints show inconsistent links with objective cognition, psychiatric symptoms, and illness insight. The Subjective Scale to Investigate Cognition in Schizophrenia (SSTICS), a validated self-report tool developed in Canada and used internationally, enables a homogeneous synthesis. **Objective:** This meta-analysis examined whether subjective complaints measured with the SSTICS are associated with objective cognition, psychiatric symptoms, and illness insight, and reviewed its factorial structure. **Method:** We conducted a meta-analysis following PRISMA guidelines. Systematic searches of PubMed, Web of Science, and Google Scholar identified studies using the SSTICS in psychiatric populations. Eligible studies compared patients to healthy controls and/or examined correlations with cognition, symptoms, or illness insight or conducted factor analyses of the SSTICS domains. **Results:** Twenty-five studies (*N* = 3,205) met criteria. Schizophrenia patients reported more cognitive complaints than controls (*d* = 0.746). No significant correlation emerged with global objective cognition (*r* = 0.105). Complaints were unrelated to positive, negative, or general symptoms but showed a moderate association with depressive symptoms (*r* = 0.300) and a small one with illness insight (*r* = 0.155). Factor analyses consistently identified 3 domains: memory, attention, and daily living. **Conclusions:** SSTICS-based complaints are substantial in schizophrenia but largely dissociated from objective cognition, reflecting impaired neurocognitive insight. Instead, they are more strongly linked to depressive symptoms, suggesting complaints reflect emotional distress rather than actual deficits. Findings support refining SSTICS subscales and extending investigations to other psychiatric populations.

## Plain Language Summary Title

Schizophrenia and Cognitive Complaints

Plain Language Summary

This paper presents a meta-analysis, which is a research method that combines results from many separate studies to provide a clearer and more reliable overall conclusion. We examined how people with schizophrenia report their own cognitive difficulties, such as problems with memory, attention or planning, and how these self-reports relate to actual performance on cognitive tests. All studies included in this meta-analysis used the same questionnaire, the Subjective Scale to Investigate Cognition in Schizophrenia (SSTICS), to measure subjective cognitive complaints. Across studies, people with schizophrenia reported significantly more thinking and memory difficulties than people without the illness. However, these complaints did not strongly match objective test results, meaning that how patients feel about their cognitive abilities often does not reflect how they perform on standardized tests. We also found that these complaints were not closely tied to positive or negative psychotic symptoms. Instead, they showed a moderate association with depressive symptoms: individuals who felt more depressed tended to report more cognitive problems. A small association was also found between cognitive complaints and insight into illness. Together, these findings suggest that subjective cognitive complaints in schizophrenia may reflect emotional distress rather than true awareness of cognitive deficits. This highlights the importance of evaluating both patient experiences and objective cognitive skills when planning treatment, rehabilitation and support.

## Résumé

**Contexte:**

Les plaintes cognitives subjectives, c’est-à-dire la conscience des déficits neurocognitifs, correspondent à la perception que les patients ont de leur fonctionnement cognitif. Dans le cas de la schizophrénie, ces plaintes montrent des liens variables avec la cognition objective, les symptômes psychiatriques et la conscience de la maladie. La Subjective Scale to Investigate Cognition in Schizophrenia (SSTICS), un outil d’autodéclaration validé, mis au point au Canada et utilisé à l’échelle internationale, permet d’en offrir une synthèse homogène.

**Objectif:**

La présente méta-analyse visait à déterminer si les plaintes subjectives mesurées avec la SSTICS sont associées à la cognition objective, aux symptômes psychiatriques et à la conscience de la maladie, et à en examiner la structure factorielle.

**Méthode:**

Une méta-analyse a été réalisée conformément aux lignes directrices PRISMA. Des recherches systématiques dans PubMed, Web of Science et Google Scholar ont permis de recenser des études menées auprès de populations psychiatriques ayant utilisé la SSTICS. Les études admissibles comparaient les patients à des témoins sains et examinaient les corrélations avec la cognition, les symptômes ou la conscience de la maladie, ou comportaient des analyses factorielles des domaines de la SSTICS.

**Résultats:**

Vingt-cinq études (*N* = 3 205) répondaient aux critères. Les patients atteints de schizophrénie rapportaient davantage de plaintes cognitives que les témoins (*d* = 0,746). Aucune corrélation significative n’a été observée avec la cognition objective globale (*r* = 0,105). Les plaintes n’étaient pas associées aux symptômes positifs, négatifs ou généraux, mais présentaient une corrélation modérée avec les symptômes dépressifs (*r* = 0,300) et une corrélation faible avec la conscience de la maladie (*r* = 0,155). L’analyse des facteurs a permis de relever trois domaines constants : la mémoire, l’attention et la vie quotidienne.

**Conclusions:**

Les plaintes évaluées à l’aide de la SSTICS sont fréquentes chez les personnes atteintes de schizophrénie, mais demeurent généralement indépendantes de la cognition objective, ce qui suggère une altération de la conscience des déficits neurocognitifs. Elles sont toutefois plus étroitement liées aux symptômes dépressifs, indiquant que ces plaintes reflètent davantage la détresse émotionnelle plutôt que de véritables déficits. Ces résultats plaident en faveur du raffinement des sous-échelles de la SSTICS et de l’élargissement des recherches à d’autres populations psychiatriques.

## Introduction

In psychiatry, the concept of insight refers to an individual's capacity to recognize the presence of a psychiatric disorder and to acknowledge the pathological nature of their own symptoms. This notion, central to clinical assessment, has been primarily studied in the context of schizophrenia, a disorder in which the lack of self-awareness is particularly pronounced.^1^ Research on insight in schizophrenia initially focused mainly on the recognition of positive psychotic symptoms, such as hallucinations and delusional ideas.^2^ Several studies have shown that the patients’ lack of insight undermines the therapeutic alliance and treatment adherence, 2 factors associated with a long-term favourable clinical prognosis.^3^

Concurrently, there has been increasing scientific attention directed towards another key dimension of schizophrenia, namely their neurocognitive deficits. These impairments affect several domains, such as attention, working memory, episodic memory (both verbal and visual), processing speed and executive functions.^4^ These cognitive deficits are present in 70% to 75% of schizophrenia cases, highlighting their high prevalence in this disorder.^5^ Overall, the cognitive deficits associated with schizophrenia are 1 to 1.2 standard deviation below that of the general population.^6^ Although psychiatric symptoms are related to cognitive performance, current evidence suggests that cognition, rather than symptom severity, is the strongest predictor of functional outcomes in schizophrenia.^7^ Noteworthy, several studies have shown that these cognitive deficits predict a larger proportion of the variance in social and occupation functioning, as compared to the proportion of the variance in functional outcomes explained by psychiatric (e.g., positive and negative) symptoms. These results thus emphasize the crucial clinical importance of cognitive deficits in the course, prognosis and rehabilitation of the disorder.^8,9^ In this context, the extent to which patients are aware of these difficulties represents a critical clinical concern.

Over the past decade, an increasing number of studies have attempted to document patients’ awareness of their cognitive deficits, that is, their ability to perceive and self-assess their own neuropsychological performance. This awareness, which can be referred to as neurocognitive insight, is distinct from general insight into the disorder. To investigate neurocognitive insight, researchers have used various self-report questionnaires measuring subjective cognitive complaints, which are then compared to objective measures obtained through standardized neuropsychological tests. Such questionnaires include the Cognitive Failures Questionnaire (CFQ),^10^ the Prospective and Retrospective Memory Questionnaire (PRMQ),^11^ the Schizophrenia Cognition Rating Scale (SCoRS),^12^ the Measure of Insight into Cognition-Self-Report (MIC-SR)^13^ and Subjective Scale to Investigate Cognition in Schizophrenia (SSTICS), the latter having been developed by our research team.^14^ Using these questionnaires, several studies have been performed to investigate subjective cognition, cognitive complaints or neurocognitive insight in schizophrenia. Unfortunately, findings across these studies have been mixed. Some reported no significant correlation between subjective and objective cognition,^15,16^ while others reported modest but significant associations.^14,17,18^ Likewise, research has sought to determine whether cognitive awareness is related to psychiatric symptoms and insight into illness, with results being heterogeneous,^19^ especially in the case of positive symptoms, with studies showing both positive and negative associations.^20^

These issues were systematically examined by Potvin and colleagues, who published a meta-analysis synthesizing the existing knowledge on the awareness of neurocognitive deficits in schizophrenia.^21^ This meta-analysis included 22 studies, totalling 1,609 schizophrenia patients and 294 healthy controls. The results showed that patients reported significantly more cognitive complaints than controls (Cohen's *d* = 0.546), but the correlation between subjective and objective cognition was small (*r* = 0.173). The strongest correlation was found for executive functions (*r* = 0.334); however, this result was based on a small subset of studies. Subjective complaints were significantly associated with depressive symptoms (*r* = 0.314), with no associations being found with positive and negative symptoms. Finally, a weak association with illness insight (*r* = 0.185) was observed. Despite its strengths, this meta-analysis had a few limitations. One of the main concerns was the heterogeneity of the instruments used to measure subjective cognition across studies. The meta-analysis has included studies based on various scales (CFQ, PRMQ, SCoRS, MIC-SR, SSTICS), which do not assess the same cognitive dimensions or use the same dimension definitions. Notably, a sub-analysis revealed that the strength of correlations between subjective and objective cognition seemed to be influenced by the choice of the scale. This methodological diversity made it difficult to compare results and to draw general conclusions about cognitive awareness in schizophrenia.

Among the instruments used, the SSTICS has been one of the most frequently employed and has demonstrated very good to excellent psychometric qualities.^21^ Specifically developed for patients with schizophrenia, this self-report scale assesses subjective complaints across several cognitive domains, including attention, working memory, verbal and visual memory, executive functions, language and praxia, some of which overlap with the 6 core domains identified by the Measurement and Treatment Research to Improve Cognition in Schizophrenia (MATRICS) consensus group.^22^ Although this result must be considered as preliminary, studies using the SSTICS included in the meta-analysis had reported stronger correlations with objective performance than those using other instruments.^21^ At the time the original meta-analysis was published, the number of studies based exclusively on the SSTICS was too limited to allow for a targeted review. Since 2014, the SSTICS, a Canadian scale now used in 12 countries, has been translated, validated and widely adopted, leading to a significant increase in the number of publications based solely on this scale.^23^ This development creates the opportunity for a new meta-analysis, methodologically more homogeneous, focusing exclusively on the SSTICS. Simultaneously, there has been a gradual expansion in the use of the SSTICS to other psychiatric disorders, notably bipolar disorder.^24^ This expansion reflects the growing recognition of the clinical validity of this tool beyond the strict framework of schizophrenia.

In this context, the present meta-analysis aims to update and refine the findings reported in 2014 by focusing exclusively on studies that have used the SSTICS as a measure of subjective cognition in patients with schizophrenia.^21^ Specifically, this meta-analysis seeks to address 4 primary objectives: (A) to determine the extent to which patients with schizophrenia report significant subjective cognitive complaints, compared to healthy subjects; (B) to evaluate the strength of correlation between these complaints and objective cognitive performance, and to identify the cognitive domains displaying the strongest correlations; (C) to examine whether subjective cognitive complaints are associated with specific psychiatric symptoms, particularly positive, negative or depressive symptoms and (D) to assess whether these complaints are related to insight into illness. Finally, a secondary objective was to review the factor analyses having been performed using the SSTICS in order to determine if the factor structure of the scale is consistent across studies^25,26^ and if its structure converges with the cognitive domains that were meant to be measured when the SSTICS was initially created.^14^ By focusing exclusively on studies utilizing the SSTICS, this meta-analysis aims to reduce the methodological heterogeneity associated with the choice of the measurement instrument and to improve comparability across studies, in the hope of providing more reliable estimates of the relationships between subjective cognition, objective cognition and clinical symptoms in schizophrenia.

Based on our previous meta-analysis using a mix of subjective cognitive complaint scales,^21^ we hypothesized that (1) patients with schizophrenia would report significantly greater subjective cognitive complaints than healthy controls; (2) subjective complaints would show weak associations with objective cognitive performance but moderate associations with depressive symptoms and (3) subjective complaints would display small but significant correlations with illness insight.

## Methods

### Selection Procedures

#### Literature Search

A literature search was conducted using the PubMed, Web of Science and Google Scholar databases, with the following keywords: schizophrenia OR psychosis AND cognitive insight OR cognitive complaints OR subjective cognition OR SSTICS. We included studies published before April 2025. To request additional data and clarify methodological details for inclusion in our meta-analysis, we obtained access to relevant thesis and contacted their authors to request additional data and clarify methodological details for inclusion in our meta-analysis on the SSTICS.

#### Selection Criteria

The studies were selected based on the following inclusion criteria: (i) they included patients diagnosed with schizophrenia spectrum or psychotic disorders, established using recognized diagnostic criteria; and (2) they included an assessment of subjective cognition using the SSTICS. Studies using the SSTICS were included as long as they examined one of the following questions: (i) the comparison of cognitive complaints between patients and controls; (ii) the examination of the correlation between subjective and objective cognition in a psychiatric population; (iii) the examination of the correlation between subjective cognition and psychiatric symptoms (including insight into illness) and (iv) the factor structure of the SSTICS domains. For this latter objective, psychiatric symptoms had to be assessed with a validated scale or interview for the study to be included. Studies were excluded if they used a scale or an interview other than the SSTICS to assess subjective cognition. Studies solely examining the relationship between the SSTICS and functional outcomes were excluded. Studies with incomplete data were also excluded, as well as studies using non-parametric statistics. In the case of overlapping samples between the studies, we used the most recent publication or the publication with the largest sample size. The decision to include or exclude a study was made by NW and SP, and disagreements were resolved by consensus.

#### Data Extraction

Data extraction was performed by NW and revised by SP. The following variables were extracted: Author, year of publication, sample size, SSTICS scores (mean and SD), correlation of the SSTICS with cognition and/or psychiatric symptoms, including insight into illness (depending on availability). This data extraction followed PRISMA guidelines. The PRISMA checklist is available in Supplemental Table 1.

### Statistical Analyses

The data were analysed using software Comprehensive Meta-Analysis, version 2 (Biostat, Inc., Englewood, NJ, USA), which uses the same computational algorithms as the Cochrane group, as it weights the relative importance of studies using an inverse variance method.^27^ The difference in subjective cognitive complaints between psychiatric and healthy subjects was assessed using Cohen's *d*, which corresponds to the difference in means between the 2 groups, divided by the pooled standard deviation. By convention, a Cohen's *d* of 0.2, 0.5 and 0.8 is considered *small*, *medium* and *large*, respectively.^28^ To evaluate the relationship between cognitive complaints measured by the SSTICS (total score) and objectively measured cognitive performance using validated neuropsychological tests, we used Pearson's correlation coefficient (*r*), which described the linear relationship between subjective and objective cognition in psychiatric patients. This analysis was conducted by combining all the cognitive domains reported in each of the available studies. More specifically, we calculated the average of the correlations for each task reported in each study included in the meta-analysis. In addition, we performed domain-specific analyses. As in previous meta-analyses, we examined attention, executive functions, fluency, language, learning/memory, speed of processing and working memory.^21,29,30^ The relationship between subjective cognitive complaints and psychiatric symptoms, on the one hand, and insight into illness, on the other, was also assessed using Pearson's *r*. To simplify the interpretation of the results, the direction of the correlation was considered positive when the cognitive complaints were greater and the cognitive deficits were worse, and when the cognitive complaints were greater and the psychiatric symptoms (including the lack of insight into illness) were more severe. Across studies, positive and negative symptoms were all measured with the Positive and Negative Syndrome Scale (PANSS).^31^ As for depressive symptoms, they were measured with the depression factor from the PANSS, the Calgary Depression Scale for Schizophrenia (CDSS),^32^ the Beck Depression Inventory-II^33^ or the Hospital Anxiety and Depression Scale.^34^ Insight into illness was measured by the G12 item from the PANSS (Lack of judgement and insight) or specific scales like the Birchwood Insight Scale^35^ or the Scale to Assess Unawareness of Mental Disorder.^4^

Heterogeneity between the studies was calculated using Cochran's *Q* and assessed using the *I*^2^ index.^36^ By convention, an *I*^2^ index of 25%, 50% and 75% is considered low, moderate and high heterogeneity, respectively. Given that our database exhibited significant heterogeneity (see below), we aggregated the effect sizes or correlation coefficients across studies using random-effects models (rather than fixed-effects models), as these models account for heterogeneity between the studies and provide more conservative estimates of the composite effect size. For analyses based on ≥10 studies, we assessed the possibility of publication bias using Egger's test^37^ and Kendall's tau.^38^

Secondary analyses were performed on the potential influence of SSTICS’ language of administration (Arab, English, French, etc.), clinical stage (first episode of psychosis, established schizophrenia) and the scale used to assess depressive symptoms (CDSS, PANSS). Finally, during the conduct of the meta-analysis, we noticed that some authors did not report the Pearson's *r* when the relationship between objective and subjective cognition was non-significant. To address this issue, we replaced unreported values with the lower bound of the confidence interval of the composite effect size. This is a recognized valid approach.^39^

## Results

### Included Studies

**Figure 1.**
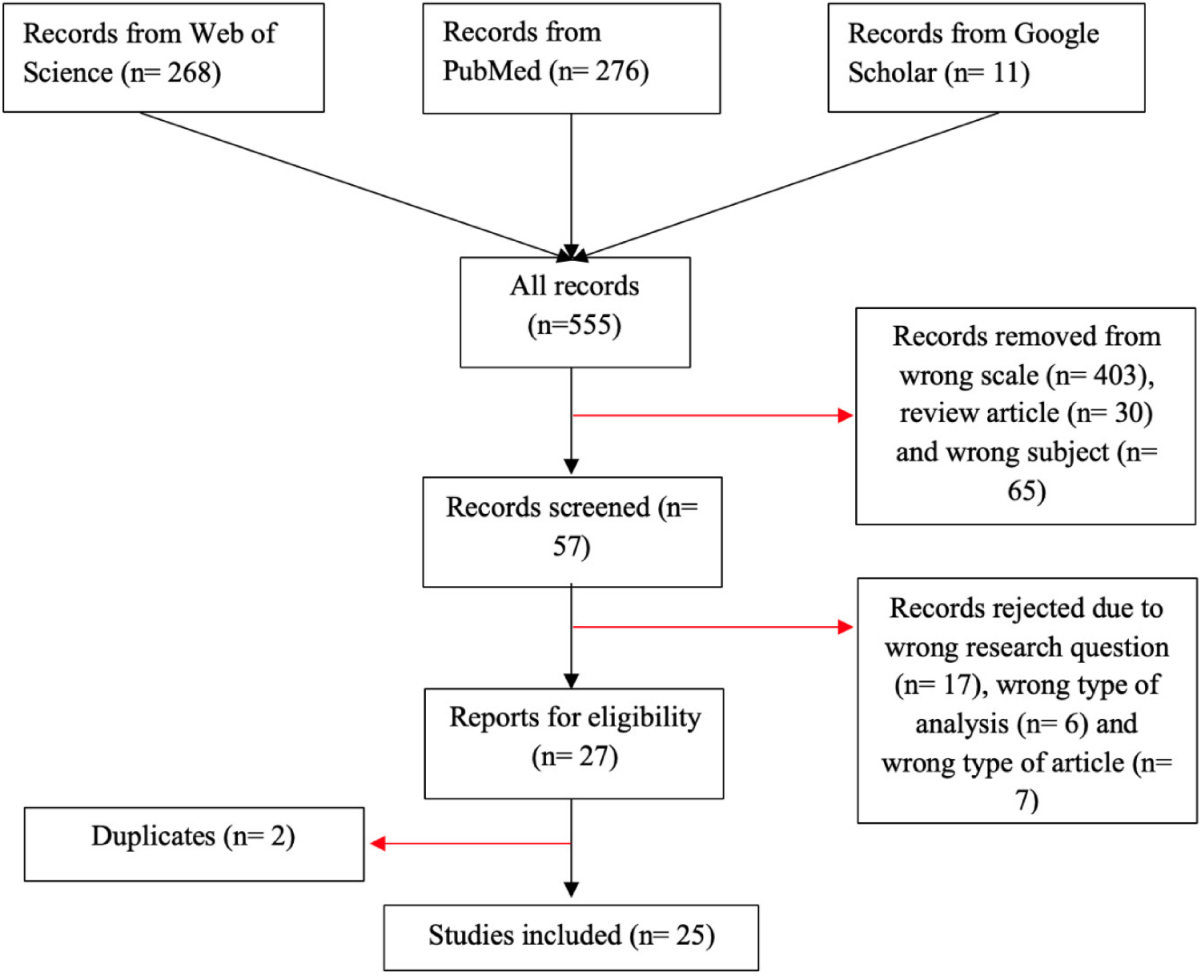
PRISMA flowchart of study selection.

The initial search yielded 714 articles: 360 from PubMed, 338 from Web of Science and 16 from Google Scholar. After the first screening of abstracts, 657 records were excluded because they used a cognitive scale other than the SSTICS (*n* = 547), were review articles (*n* = 40) or addressed wrong topics (*n* = 70). A total of 57 full-text articles were then assessed for eligibility. Of these, 29 were excluded for not meeting the inclusion criteria (unrelated research question, *n* = 17; analysis not usable for the present meta-analysis, *n* = 6; topic outside the study scope, *n* = 6). In addition, 2 duplicate articles from the same author were identified at this stage. Ultimately, 25 studies were retained and included in the meta-analysis^14,16,25,26,40–58^(see Figure 1 for the flowchart and Table 1 for the description of the included studies).

**Table 1.** Description of the Studies Included in the Meta-Analysis.

Authors	Sample	Comparison with Controls?	Correlation with Objective Cognition?	Correlation with the PANSS?	Correlation with Depressive Symptoms?	Correlation with Insight into Illness?	Factorial Analysis?	Language of SSTICS Administration	Clinical Stage
Al Mugaddam, 2025	126 mixed	Yes	No	No	No	No	No	Emirati Arabic	Not specified
Baliga, 2020	100 SCZ	No	No	General, positive, negative and total	PANSS	SUMD	No	Hindi and Marathi	Chronic
Bayard, 2009	101 SCZ	Yes	No	General and positive	No	No	No	French	Chronic
Cella, 2020	607 SCZ	No	No	General, positive, negative and total	No	No	Yes	English	Chronic and first-episode psychosis
Chuang, 2019	37 SCZ	No	LNNB; Receptive speech and arithmetic	No	No	No	No	Taiwanese	Chronic
Gopal, 2024	191 SCZ	No	No	No	No	No	Yes	Tamil	Chronic
Grimstad, 2025	SCZ = 91, BD = 55	Yes	MCCB; NAB Mazes	General, positive and negative	CDSS	PANSS item G12	No	Norwegian	Not specified
Haddad, 2021	180 SCZ	Yes	BACS; VM, DST, MT, LF, SF, SC, TOL	No	CDSS	No	No	Tunisian Arabic	Chronic
Haddad, 2023	120 SCZ	No	No	General, positive, negative and total	CDSS	No	No	Arabic	Chronic
Johnson, 2009	105 SCZ	No	No	Positive	CDSS	PANSS item G12	Yes	Tunisian Arabic	Chronic
Lecardeur, 2009	176 SCZ	No	No	General, positive and negative	PANSS	No	No	French	Chronic
Marucci, 2018	44 SCZ	No	Wisconsin card sorting test	Negative, positive and total	PANSS	PANSS item G12	Yes	Italian	Chronic
Potvin, 2005	76 SCZ	No	CANTAB; PAL	No	No	No	No	French	Chronic
Potvin, 2017	82 SCZ	No	No	No	No	No	Yes	French	Chronic
Prouteau, 2004	73 SCZ	No	CANTAB; MOT, PAL, SOC, RTI	No	No	No	No	French	Chronic
Prouteau, 2015	82 SCZ	Yes	No	No	No	No	No	French	Chronic
Raffard, 2020	50 SCZ	Yes	TAP	General, positive, Negative and total	BDI-II	No	No	French	Chronic
Santarelli, 2020	109 SCZ	No	No	Positive	PANSS	No	No	Italian	Not specified
Seco, 2010	46 SCZ	No	Test Barcelona, Dd, Di, Pp, Mt, Mv, Ac, Cn, C	No	No	No	No	Spanish	Chronic
Sellwood, 2013	160 SCZ	Yes	BACS: DST, SF, SC, VM, LF, TOL	Positive and negative	Hospital Anxiety and Depression Scale	BIS	No	English	Chronic
Shin, 2016	70 SCZ	No	No	No	CDSS	No	No	Korean	Chronic
Stip, 2003	114 SCZ	No	RAVLT	General and negative	CDSS	PANSS item G12	Yes	French	Chronic
Stip, 2022	198 SCZ	No	No	No	CDSS	PANSS item G12	No	French	First-episode psychosis
Stratta, 2020	131 SCZ	No	No	Negative and positive	PANSS	PANSS item G12	Yes	Italian	Chronic
Zhornitsky, 2011	81 SCZ	No	No	No	No	PANSS item G12	No	French	Not specified

*Note*. Ac = Abstracción-comprensión (Barcelona); BACS = Brief Assessment of Cognition in Schizophrenia; BD = bipolar disorder; BDI-II = Beck Depression Inventory-Second Edition; BIS = Birchwood Insight Scale; BVMT-R = Brief Visuospatial Memory Test-Revised; C = Cubos (Barcelona); CANTAB = Cambridge Neuropsychological Test Automated Battery; CDSS = Calgary Depression Scale for Schizophrenia; Cn = Clave de números (Barcelona); Dd = Dígitos directos (Barcelona); Di = Dígitos inversos (Barcelona); DST = Digit Sequencing Task (BACS); LF = Letter Fluency (BACS); LNNB = Luria-Nebraska Neuropsychological Battery; MCCB = MATRICS Consensus Cognitive Battery; MOT = Motor Screening (CANTAB); Mt = Memoria de textos (Barcelona); MT = Token Motor Task (BACS); Mv = Memoria visual (Barcelona); NAB Mazes = Neuropsychological Assessment Battery – Mazes; PAL = Paired Associates Learning (CANTAB); PANSS = Positive and Negative Syndrome Scale; Pp = Evocación categórica (Barcelona); RAVLT = Rey Auditory Verbal Learning Test; RTI = Reaction Time (CANTAB); SC = Symbol Coding (BACS); SCZ = patients with schizophrenia; SF = Semantic Fluency (BACS); SOC = Stockings of Cambridge (CANTAB); SUMD = Scale to Assess Unawareness of Mental Disorder; TAP = Test of Attentional Performance; TOL = Tower of London (BACS); VM = Verbal Memory (List Learning) (BACS).

### Cognitive Complaints

**Figure 2.**
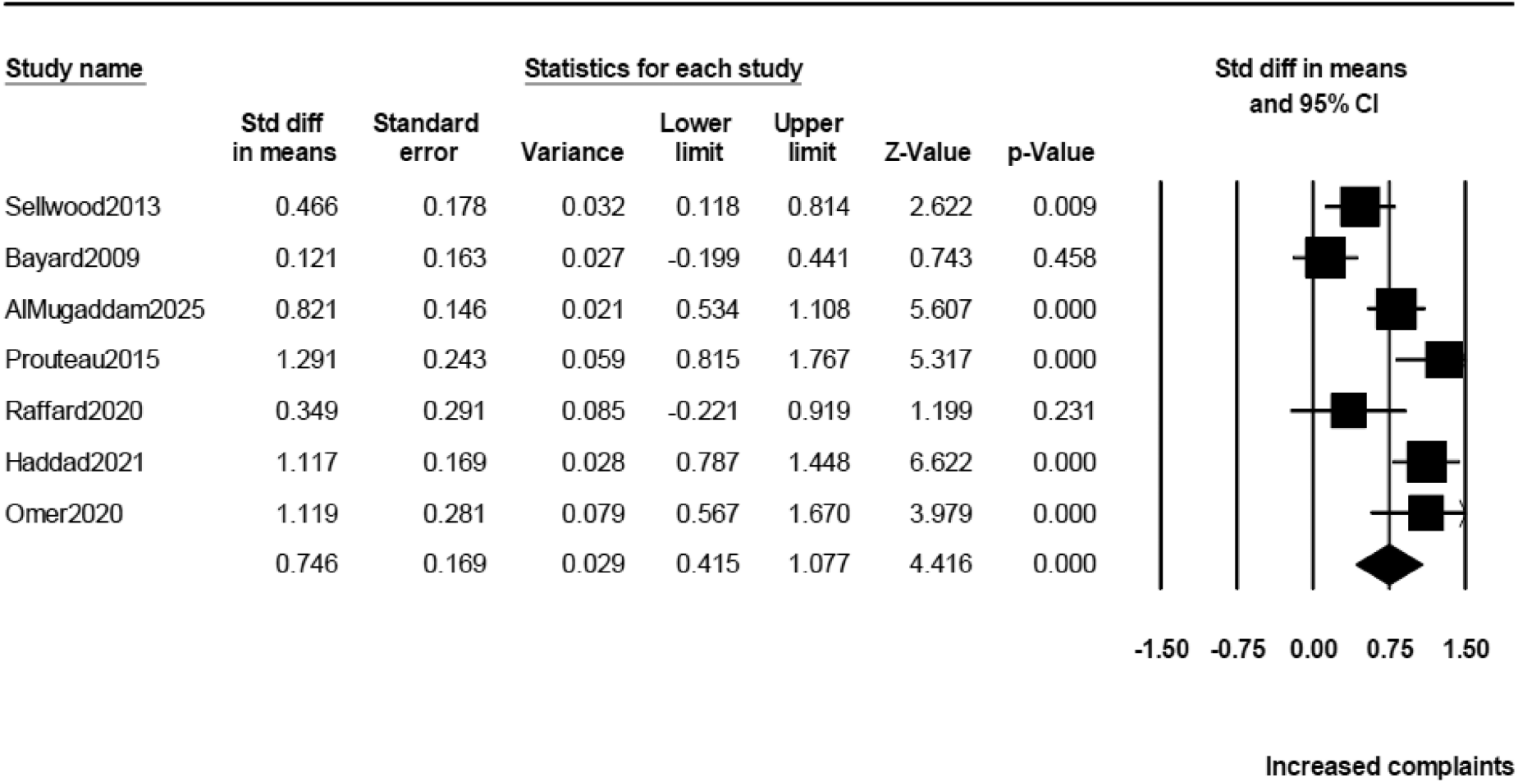
Forrest plot of the effect size estimates (Cohen's *d*) for the differences in subjective cognitive complaints (total SSTICS score) between schizophrenia patients and healthy controls. SSTICS = Subjective Scale to Investigate Cognition in Schizophrenia.

As shown in Table 2 and Figure 2, schizophrenia patients reported a significantly higher level of cognitive complaints, as assessed by the SSTICS total, compared to healthy subjects. The magnitude of this difference was moderate to large (*d* = 0.746).

**Table 2.** Meta-Analysis of the SSTICS in Schizophrenia.

Variable/Comparison	Number of Studies	Effect Size (*d* or *r*)	95% Confidence Interval	*P*-Value	*I*^2^ (%)
Differences in subjective cognitive complaints between schizophrenia patients and healthy controls					
Total SSTICS score	7	*d* = 0.746	0.415 to 1.077	0.0001	80.5
Correlation between subjective cognition and clinical variables					
Global objective cognition	10	*r* = 0.105	−0.048 to 0.253	0.180	73.0
PANSS-Positive	13	*r* = -0.010	−0.136 to 0.116	0.871	83.8
PANSS-Negative	14	*r* = 0.075	−0.006 to 0.156	0.070	62.8
PANSS-General	8	*r* = 0.075	−0.065 to 0.213	0.292	80.0
PANSS-Total	6	*r* = 0.033	−0.159 to 0.223	0.737	78.1
Depressive symptoms	13	*r* = 0.300	0.182 to 0.409	0.0001	79.5
Insight into illness^a^	9	*r* = 0.155	0.092 to 0.216	0.0001	11.8

*Note*. PANSS = Positive and Negative Syndrome Scale; SSTICS = Subjective Scale to Investigate Cognition in Schizophrenia; CI = confidence interval.

^a^ Data was analysed with a fixed-effect model due to low heterogeneity.

### Correlation Analyses

#### Global Objective Cognition

As shown in Table 2, there was no significant correlation between cognitive complaints and global objective cognition (*r* = 0.105), regardless of the cognitive domains being assessed.

Studies using the French and Other language versions of the SSTICS failed to detect significant associations with objective global cognition (see Supplemental Table 2). Likewise, clinical stage had no influence on results, since studies involving schizophrenia or established schizophrenia patients all revealed no significant associations between objective global cognition and subjective complaints.

Domain-specific analyses revealed a significant and positive association between subjective complaints and learning/memory (*r* = 0.270) as well as working memory (*r* = 0.228) (Supplemental Table 3). A non-significant association was found with fluency. Small or very small associations were observed in the case of executive functions and speed of processing. Finally, negative associations were found for attention and language.

#### Psychiatric Symptoms

**Figure 3.**
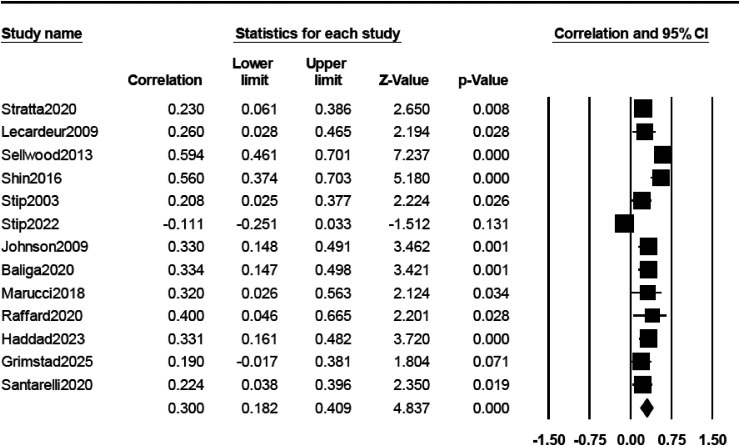
Forrest plot of the correlations between subjective cognitive complaints and depressive symptoms.

There was no significant association between cognitive complaints and positive, negative, general and total psychiatric symptoms measured with the PANSS (Table 2). Conversely, a significant, moderate and positive association was found between cognitive complaints and depressive symptoms (*r* = 0.300) (Figure 3). The association between subjective complaints and depressive symptoms was larger in studies using other scales (*r* = 0.543) than the CDSS (*r* = 0.254) or the PANSS (*r* = 0.265) (Supplemental Table 4).

A positive association was found between positive symptoms and subjective complaints when these were assessed with the English version of the SSTICS, while a negative association was found in studies using the Italian version (see Supplemental Table 5). For negative symptoms, only studies using the Italian version of the SSTICS showed a positive association between negative symptoms and subjective complaints. Conversely, all studies showed a positive association between depressive symptoms and subjective cognitive complaints, apart from the studies using the original French version of the SSTICS. For positive, negative and depressive symptoms, clinical stage (established schizophrenia, first episode of psychosis) had little or no influence on results.

#### Insight into Illness

A small-range, significant and positive association was observed between subjective complaints and insight into illness (*r* = 0.155).

#### Publication Bias

For all the correlation analyses based on ≥10 studies, no evidence of publication bias was detected, apart from a marginal association between subjective complaints and global cognition (see Supplemental Table 5 and Figure 1).

### Review of the Studies Assessing the Factor Structure of the SSTICS

First, a total of 7 factor analyses were identified. Among them, 5 were performed using all 21 items of the SSTICS. All 5 used exploratory factor analyses, except for the study by Stratta et al., who also conducted a confirmatory factor analysis (for more details, refer to Supplemental Table 6).^25^ Among the 5 studies that used the 21 items, the number of factors ranged from 4 to 6. The “Memory” and “Attention/Distractibility” factors were replicated in all analyses. The “Daily Living” factor emerged in 4 studies, while the “Executive Function” factor appeared in 3 analyses. The “Working Memory”, “Praxia”, “Language” and “Consciousness” factors were only identified in 2 studies each. In contrast, both Cella et al. and Al Mugaddam et al. identified only 1 factor, although Cella et al. employed the abbreviated 14-item SSTICS-brief version.^26,40^

## Discussion

This meta-analysis aimed to better understand subjective cognitive complaints in individuals with schizophrenia, by comparing them to healthy controls and by examining their associations with objective cognition, psychiatric symptoms and insight into illness. All studies examined subjective cognition using the SSTICS in schizophrenia, with 1 also including bipolar disorder. Results showed that schizophrenia patients reported significantly more cognitive complaints than healthy individuals (*d* = 0.746). This confirms these complaints are substantial. However, complaints were unrelated to global cognition (*r* = 0.096), showing a dissociation between perceived and measured performance. Regarding psychiatric symptoms, no significant correlations were found with positive, negative, general or total PANSS scores. In contrast, a moderate and significant association was observed with depressive symptoms (*r* = 0.303). A small but significant positive correlation was also found with insight into illness (*r* = 0.155). Clinical stage (first episode of psychosis vs. established schizophrenia) had little or no influence on results.

Despite high complaint levels, our meta-analysis found no significant correlation between subjective and global objective cognition. This dissociation supports the notion of impaired neurocognitive insight: individuals may notice cognitive problems yet lack awareness of specific deficits.^20,59^ The current results align with Chan et al., who used other scales such as the Subjective Cognitive Impairment Scale and similarly found no strong associations with objective neuropsychological tests.^60^ A previous meta-analysis also reported no link between complaints and global cognition, though it combined results from several subjective cognition scales.^21^ By focusing on a single scale here (SSTICS), the present findings suggest that the absence of association is a robust phenomenon rather than an artefact of scale heterogeneity.^20,21^ While some studies have noted domain-specific associations, such as with working memory or problem solving,^21,44^ the broader evidence indicates that subjective complaints mainly reflect generalized discomfort rather than accurate awareness of cognitive deficits.^20,45^ These results suggest that subjective complaints in schizophrenia are not valid indicators of cognitive performance but instead reflect reduced insight into neurocognitive functioning.

Although the SSTICS does not specify a precise reference period, many items ask about the frequency of current cognitive difficulties or whether certain activities can still be performed as easily as before. Consequently, the SSTICS may capture perceived changes relative to an individual's own pre-illness baseline rather than performance compared to population norms. This could explain the weak cross-sectional correlation with objective cognition, which might become stronger when longitudinal changes in cognition are examined. Future research should therefore investigate within-subject variations over time to determine whether objective cognitive improvement parallels reductions in subjective complaints.

Our meta-analysis found no significant correlation between cognitive complaints and positive or negative symptoms. Effect sizes for positive symptoms varied across studies, with some showing that greater symptom severity was linked to more complaints and others the opposite, yielding an overall effect near zero with high heterogeneity. In contrast, depressive symptoms consistently showed a moderate and significant correlation with subjective complaints (*r* ≈ 0.30), suggesting that mood disturbances strongly influence how patients perceive cognitive difficulties.^61^ This supports the idea that complaints reflect emotional distress rather than actual neurocognitive impairment,^21^ consistent with reduced neurocognitive insight in schizophrenia. Supporting this view, studies using scales other than the SSTICS have also found links between depressive symptoms and subjective complaints in schizophrenia.^62^ Likewise, studies investigating subjective and objective cognition in major depression using self-report scales other than the SSTICS have also shown positive associations between subjective cognitive complaints and depressive symptoms severity.^60^ As such, our results are not unexpected and suggest either that a greater awareness of cognitive deficits elicits depressive feelings and/or that depressed individuals are more likely to complain about different conditions, including cognitive impairments.

A small but significant correlation was found between subjective cognitive complaints and insight into illness (*r* ≈ 0.15), suggesting that while some overlap exists, these domains are largely independent. Thus, awareness of illness and awareness of one's cognitive deficits operate separately: a patient may recognize having a disorder without perceiving cognitive problems or feel cognitively impaired while denying psychiatric illness. This pattern is consistent with Baliga et al., who showed neurocognitive insight to be independent of clinical insight and psychotic symptoms, and with Holthausen et al., who found that cognitive and clinical insight may co-occur but remain distinct constructs linked to broader meta-cognitive dysfunction.^41,62^ Similarly, Potvin et al. reported weak correlations with insight into illness across various subjective cognition scales, indicating that this dissociation is not due to scale heterogeneity but reflects distinct meta-cognitive domains.^21^ Our focused analysis using the SSTICS confirms the robustness of this separation and highlights the need to evaluate these domains of insight independently in research and clinical settings. The present findings reinforce the value of this concept and ongoing efforts in the meta-cognitive literature to clarify forms of impaired insight in schizophrenia.^63,64^ Although our results suggest that clinical insight and neurocognitive insight are relatively independent constructs, insight into illness has also been linked to depressive symptoms in schizophrenia, with greater awareness being associated with higher levels of distress.^65^ Insofar as the awareness of one's own difficulties may elicit distress, future research will need to determine if there is an additive effect (or not) between clinical and neurocognitive insight on the emergence of depressive symptoms in schizophrenia.

The SSTICS was initially designed to assess 6 cognitive domains: attention, working memory, verbal and visual memory, executive functions, language and praxia.^14^ Yet, factorial analyses in the literature consistently identify only 3 robust domains: memory, attention and daily living. This discrepancy raises the question of whether it reflects scale design or reporting difficulties in psychosis. Some domains, such as language and praxia, may be under-represented, since the SSTICS includes only 2 related items. Their failure to load on distinct factors could mean that the items were unproperly chosen or that patients did not perceive them as separate constructs. Another possibility is limited statistical power due to the small number of items for these domains. The consistent emergence of memory and attention supports partial validity of the scale. Finally, some analyses have reported a “daily living” factor, but it combined praxia, memory and/or executive function items, suggesting that certain constructs in the SSTICS may require refinement. Although most factorial analyses converge towards 5 or 6 domains,^14,42,46,50^ a few studies have instead reported a single overarching factor, notably with the SSTICS-brief^26^ and in 1 recent validation study,^40^ further contributing to the heterogeneity in the literature.

Social cognition was not examined in the present meta-analysis. The SSTICS does not include items related to social cognitive domains such as emotion recognition or theory of mind, making it unsuitable for assessing subjective social cognitive deficits. Recently, specific self-report instruments have been developed for this purpose, such as the Subjective Social Cognitive Impairment Scale,^66^ which could help clarify how individuals perceive social cognitive difficulties in schizophrenia. Future studies should investigate whether subjective social cognitive complaints relate to functional outcomes and objective social cognition measures.

A key methodological strength of the present meta-analysis is using a single, uniform measure (SSTICS), across all studies, minimizing the heterogeneity of our previous meta-analysis. The SSTICS has been translated and validated in diverse linguistic and cultural settings, including Italian,^25^ Arabic,^40^ Tunisian Arabic^14^ and adaptations into Pakistani/Urdu, Hindi, Marathi, Lithuanian, Serbian, German, Romanian, Sinhala and Russian.^40^ These cross-cultural validations, involving translations into at least 8 non-English languages, contrast with many other subjective cognition scales that were developed and normed primarily within English-speaking contexts^10,13^ and may be susceptible to linguistic and cultural biases.^67^ This suggests that the patterns we observed, namely significant subjective complaints uncorrelated with objective deficits, are unlikely to stem from cultural or linguistic biases. The observed associations across culturally distinct samples enhance the external validity of the SSTICS and underscore its utility in international research on neurocognitive insight in schizophrenia. Sub-analyses were performed on the language of administration of the SSTICS based on small numbers of studies per language. Although results of these exploratory analyses suggest potential differences between languages, it must be highlighted that we did not observe inflated results in the studies using the original version of the scale (e.g., in French). As such, these results show that the literature is not plagued by a “developer bias”.

Although exclusive SSTICS use reduced variability, a key limitation of this meta-analysis is the small number of studies available for each sub-analysis. In particular, we did not have many studies to examine the different cognitive domains separately (executive functions, attention, memory), which may not show the same magnitude of association with subjective cognitive complaints. Indeed, some studies suggest that self-perceived difficulties in planning, organizing and monitoring behaviour may reflect real-world executive deficits more reliably than general complaints.^25,67^ To address this critical question, we performed exploratory sub-analyses and found that subjective complaints were significantly associated with deficits in learning/memory and working memory. Another limitation is that we observed a publication bias for the analysis on the potential association between the SSTICS and global cognitive performance. Visual inspection of the funnel plot in Supplemental Figure 2 counter-intuitively suggests that studies with positive findings may have not been published, meaning that the potential association between subjective and objective cognition in schizophrenia may have been under-estimated. Also, the present meta-analysis was not pre-registered, which may limit transparency and reproducibility. Nonetheless, all procedures were conducted in accordance with PRISMA guidelines. Another limitation concerns the absence of data on healthy controls’ self-assessment accuracy. Only 1 included study assessed correlations between subjective complaints and objective memory among healthy participants and found a significant relationship (*r* = −0.43, *P* = 0.03), suggesting some preserved cognitive insight in non-clinical samples. However, a meta-analysis in older adults also reported small associations between subjective and objective memory.^68^ Moreover, recent work has confirmed that healthy individuals exhibit only moderate accuracy in self-assessing their cognitive performance, with typical correlations around *r* = 0.30–0.40.^20,69^ Future research should directly compare schizophrenia patients and controls to clarify whether the association between subjective and objective cognition is weaker in schizophrenia or not. Only a few studies examined associations between subjective cognition and functional outcomes, preventing quantitative synthesis. Nevertheless, preliminary evidence suggests that subjective cognitive complaints may relate to real-world functioning,^47,51^ warranting further systematic investigation. A final limitation concerns potential inter-rater variability in PANSS administration and scoring across sites and languages, which could contribute to between-study heterogeneity.

If we address clinicians, our meta-analysis shows that subjective cognitive complaints often fail to correlate with objective neuropsychological test results, particularly in psychotic contexts. This underlines the importance of listening to patient reports rather than dismissing them when results appear uncorrelated. Furthermore, subjective complaints, especially when associated with depressive symptoms, can significantly affect quality of life, distress and motivation, even in the absence of objective deficits. Related work by Australian authors^70^ emphasizes the value of systematic cognitive screening, enabling early identification of strengths and weaknesses in psychosis to personalize care and enhance engagement. One could therefore suggest placing greater emphasis on combining formal cognitive assessments with patient reports, tailoring interventions to subjective experiences and fostering engagement by acknowledging the cognitive difficulties experienced by individuals.

The aim of this meta-analysis was to determine whether subjective cognitive complaints in schizophrenia reflect objective deficits, psychiatric symptoms or clinical insight. Overall, patients reported substantial complaints, but these did not correlate with objective performance; instead, they were linked to depressive symptoms, indicating a dissociation between perception and reality. This suggests that complaints reflect psychological distress rather than true awareness of cognitive dysfunction, highlighting impaired neurocognitive insight. Future research should assess whether this dissociation holds across all domains or if certain areas, such as episodic and working memory, show stronger associations. Future research should specifically assess the relationship between subjective cognitive complaints and functional outcomes or functional decline, to determine whether perceived cognitive difficulties contribute to daily functioning independently of objective cognitive deficits. As the SSTICS is increasingly used in diverse cultural contexts, meta-analyses should examine the influence of cultural and contextual factors. Moreover, since nearly all studies focused on schizophrenia, extending investigations to other psychiatric populations is essential. Finally, revisions of the SSTICS could expand under-represented subscales to better capture certain domains and ensure a more balanced factorial structure.

## Supplemental Material

sj-docx-1-cpa-10.1177_07067437261425086 - Supplemental material for Cognitive Complaints in Schizophrenia: A Meta-Analysis of Studies Using the Subjective Scale to Investigate Cognition in Schizophrenia (SSTICS): Les plaintes cognitives dans la schizophrénie : Une méta-analyse des études utilisant la *Subjective Scale To Investigate Cognition in Schizophrenia* (SSTICS) Supplemental material, sj-docx-1-cpa-10.1177_07067437261425086 for Cognitive Complaints in Schizophrenia: A Meta-Analysis of Studies Using the Subjective Scale to Investigate Cognition in Schizophrenia (SSTICS): Les plaintes cognitives dans la schizophrénie : Une méta-analyse des études utilisant la *Subjective Scale To Investigate Cognition in Schizophrenia* (SSTICS) by Naomi White, Stéphane Potvin, André Do and Emmanuel Stip in The Canadian Journal of Psychiatry
