# Supplementary material for: Cognitive Complaints in Schizophrenia: A Meta-Analysis of Studies Using the Subjective Scale to Investigate Cognition in Schizophrenia (SSTICS): Les plaintes cognitives dans la schizophrénie : Une méta-analyse des études utilisant la Subjective Scale To Investigate Cognition in Schizophrenia (SSTICS)
Source: Can J Psychiatry. 2026 Mar 3:07067437261425086. Online ahead of print. doi: 10.1177/07067437261425086 (PMC12956623; doi:10.1177/07067437261425086)
Supplement: sj-docx-1-cpa-10.1177_07067437261425086 - Supplemental material for Cognitive Complaints in Schizophrenia: A Meta-Analysis of Studies Using the Subjective Scale to Investigate Cognition in Schizophrenia (SSTICS): Les plaintes cognitives dans la schizophrénie : Une méta-analyse des études utilisant l [file sj-docx-1-cpa-10.1177_07067437261425086.docx]

**Supplementary Table 1: PRISMA Checklist**

**Table S1.** PRISMA checklist

| Section/topic | # | Checklist item | Reported on page # |
| --- | --- | --- | --- |
| TITLE | | |  |
| Title | 1 | Identify the report as a systematic review. meta-analysis. or both. | 1 |
| ABSTRACT | | |  |
| Structured summary | 2 | Provide a structured summary including. as applicable: background; objectives; data sources; study eligibility criteria. participants. and interventions; study appraisal and synthesis methods; results; limitations; conclusions and implications of key findings; systematic review registration number. | 2 |
| INTRODUCTION | | |  |
| Rationale | 3 | Describe the rationale for the review in the context of what is already known. | 3 to 5 |
| Objectives | 4 | Provide an explicit statement of questions being addressed with reference to participants. interventions. comparisons. outcomes. and study design (PICOS). | 6 |
| METHODS | | |  |
| Protocol and registration | 5 | Indicate if a review protocol exists. if and where it can be accessed (e.g.. Web address). and. if available. provide registration information including registration number. | 8 |
| Eligibility criteria | 6 | Specify study characteristics (e.g.. PICOS. length of follow-up) and report characteristics (e.g.. years considered. language. publication status) used as criteria for eligibility. giving rationale. | 7 |
| Information sources | 7 | Describe all information sources (e.g.. databases with dates of coverage. contact with study authors to identify additional studies) in the search and date last searched. | 7 |
| Search | 8 | Present full electronic search strategy for at least one database. including any limits used. such that it could be repeated. | 7 |
| Study selection | 9 | State the process for selecting studies (i.e.. screening. eligibility. included in systematic review. and. if applicable. included in the meta-analysis). | 7 |
| Data collection process | 10 | Describe method of data extraction from reports (e.g.. piloted forms. independently. in duplicate) and any processes for obtaining and confirming data from investigators. | 8 |
| Data items | 11 | List and define all variables for which data were sought (e.g.. PICOS. funding sources) and any assumptions and simplifications made. | 7 |
| Risk of bias in individual studies | 12 | Describe methods used for assessing risk of bias of individual studies (including specification of whether this was done at the study or outcome level). and how this information is to be used in any data synthesis. | N/A |
| Summary measures | 13 | State the principal summary measures (e.g.. risk ratio. difference in means). | 8-9 |
| Synthesis of results | 14 | Describe the methods of handling data and combining results of studies. if done. including measures of consistency (e.g.. I^2^) for each meta-analysis. | 9 |
| Risk of bias across studies | 15 | Specify any assessment of risk of bias that may affect the cumulative evidence (e.g.. publication bias. selective reporting within studies). | 9 |
| Additional analyses | 16 | Describe methods of additional analyses (e.g.. sensitivity or subgroup analyses. meta-regression). if done. indicating which were pre-specified. | 9 |

| RESULTS | | |  |
| --- | --- | --- | --- |
| Study selection | 17 | Give numbers of studies screened. assessed for eligibility. and included in the review. with reasons for exclusions at each stage. ideally with a flow diagram. | 10 |
| Study characteristics | 18 | For each study. present characteristics for which data were extracted (e.g.. study size. PICOS. follow-up period) and provide the citations. | 10 and Supplementary material |
| Risk of bias within studies | 19 | Present data on risk of bias of each study and. if available. any outcome level assessment (see item 12). | N/A |
| Results of individual studies | 20 | For all outcomes considered (benefits or harms). present. for each study: (a) simple summary data for each intervention group (b) effect estimates and confidence intervals. ideally with a forest plot. | Figure 1 and Figure 2 |
| Synthesis of results | 21 | Present results of each meta-analysis done. including confidence intervals and measures of consistency. | 10 and table 1 |
| Risk of bias across studies | 22 | Present results of any assessment of risk of bias across studies (see Item 15). | N/A |
| Additional analysis | 23 | Give results of additional analyses, if done (e.g.. sensitivity or subgroup analyses. meta-regression [see Item 16]). | 9 |
| DISCUSSION | | |  |
| Summary of evidence | 24 | Summarize the main findings including the strength of evidence for each main outcome; consider their relevance to key groups (e.g.. healthcare providers. users. and policy makers). | 12-13 |
| Limitations | 25 | Discuss limitations at study and outcome level (e.g.. risk of bias). and at review-level (e.g.. incomplete retrieval of identified research. reporting bias). | 15-16 |
| Conclusions | 26 | Provide a general interpretation of the results in the context of other evidence. and implications for future research. | 16 |
| FUNDING | | |  |
| Funding | 27 | Describe sources of funding for the systematic review and other support (e.g.. supply of data); role of funders for the systematic review. | 17 |

From: Moher D. Liberati A. Tetzlaff J. Altman DG. The PRISMA Group (2009). Preferred Reporting Items for Systematic Reviews and Meta-Analyses: The PRISMA Statement. PLoS Med 6(7): e1000097. doi:10.1371/journal.pmed1000097

For more information. visit: www.prisma-statement.org.

**Supplementary Table 2: Secondary analyses on language (SSTICS) and clinical stage**

| Moderator variable | Studies | Effect size (r) | p-value | 95% confidence interval | Heterogeneity |
| --- | --- | --- | --- | --- | --- |
| Global cognition – Language | | | | | |
| Arab | 1 | 0.340 | 0.0001 | 0.171 to 0.489 | --- |
| English | 1 | -0.006 | 0.950 | -0.191 to 0.180 | --- |
| French | 4 | 0.175 | 0.234 | -0.113 to 0.436 | Q=13.4; p=0.004; I^2^=77.6% |
| Italian | 1 | -0.045 | 0.773 | -0.337 to 0.255 | --- |
| Other | 3 | -0.011 | 0.942 | -0.300 to 0.280 | Q=6.6; p=0.037; I^2^=69.6% |
| Global cognition – Clinical stage | | | | | |
| Established SCZ | 9 | 0.109 | 0.209 | -0.062 to 0.274 | Q=32.5; p=0.0001; I^2^=75.4% |
| SCZ | 1 | 0.048 | 0.679 | -0.178 to 0.269 | --- |
| Positive symptoms – Language | | | | | |
| Arab | 2 | 0.051 | 0.777 | -0.295 to 0.386 | Q=7.2; p=0.007; I^2^=86.1% |
| English | 3 | 0.183 | 0.001 | 0.073 to 0.290 | Q=4.5; p=0.107; I^2^=55.3% |
| French | 3 | -0.110 | 0.124 | -0.247 to 0.030 | Q=0.1; p=0.942; I^2^=0.0% |
| Italian | 3 | -0.289 | 0.037 | -0.521 to -0.018 | Q=10.1; p=0.006; I^2^=80.2% |
| Other | 2 | 0.105 | 0.150 | -0.038 to 0.245 | Q=0.4; p=0.514; I^2^=0.0% |
| Positive symptoms – Clinical stage | | | | | |
| Established SCZ | 9 | 0.027 | 0.654 | -0.092 to 0.146 | Q=25.6; p=0.001; I^2^=68.7% |
| FEP | 1 | 0.182 | 0.0001 | 0.081 to 0.280 | --- |
| SCZ | 3 | -0.175 | 0.390 | -0.522 to 0.222 | Q=24.6; p=0.0001; I^2^=91.9% |
| Negative symptoms – Language | | | | | |
| Arab | 2 | 0.093 | 0.326 | -0.093 to 0.272 | Q=2.0; p=0.161; I^2^=49.2% |
| English | 3 | -0.015 | 0.683 | -0.086 to 0.057 | Q=0.2; p=0.915; I^2^=0.0% |
| French | 4 | 0.064 | 0.617 | -0.184 to 0.303 | Q=13.4; p=0.004; I^2^=77.7% |
| Italian | 3 | 0.215 | 0.011 | 0.050 to 0.369 | Q=3.7; p=0.156; I^2^=46.1% |
| Other | 2 | 0.020 | 0.841 | -0.176 to 0.215 | Q=1.9; p=0.171; I^2^=46.8% |
| Negative symptoms – Clinical stage | | | | | |
| Established SCZ | 9 | 0.093 | 0.087 | -0.014 to 0.198 | Q=20.7; p=0.008; I^2^=61.4% |
| FEP | 1 | 0.001 | 0.985 | -0.102 to 0.104 | --- |
| SCZ | 4 | 0.061 | 0.546 | -0.136 to 0.254 | Q=12.2; p=0.007; I^2^=75.5% |
| Depressive symptoms – Language | | | | | |
| Arab | 2 | 0.331 | 0.0001 | 0.208 to 0.443 | Q=0.0; p=0.993; I^2^=0.0% |
| English | 1 | 0.594 | 0.0001 | 0.461 to 0.701 | --- |
| French | 4 | 0.166 | 0.163 | -0.068 to 0.383 | Q=14.2; p=0.003; I^2^=78.9% |
| Italian | 3 | 0.242 | 0.0001 | 0.128 to 0.349 | Q=0.4; p=0.839; I^2^=0.0% |
| Other | 3 | 0.366 | 0.002 | 0.143 to 0.554 | Q=7.5; p=0.024; I^2^=73.2% |
| Depressive symptoms – Clinical state | | | | | |
| Established SCZ | 10 | 0.361 | 0.0001 | 0.265 to 0.450 | Q=21.9; p=0.009; I^2^=58.9% |
| FEP | 1 | -0.111 | 0.131 | -0.251 to 0.033 | --- |
| SCZ | 2 | 0.209 | 0.003 | 0.071 to 0.339 | Q=0.1; p=0.803; I^2^=0.0% |

FEP= first episode of psychosis; SCZ= schizophrenia

**Supplementary Table 3: Correlations between the SSTICS and cognitive domains**

| Domain | Studies | Effect size (r) | P-value | 95% Confidence interval | Heterogeneity |
| --- | --- | --- | --- | --- | --- |
| Attention | 2 | -0.108 | 0.605 | -0.477 to 0.293 | Q=3.9; p=0.047; I^2^=74.7% |
| Executive functions | 5 | 0.098 | 0.228 | -0.061 to 0.252 | Q=9.3; p=0.053; I^2^=57.2% |
| Fluency | 3 | 0.213 | 0.082 | -0.027 to 0.431 | Q=7.6; p=0.022; I^2^=73.8% |
| Language | 1 | -0.350 | 0.033 | -0.605 to -0.029 | --- |
| Learning / memory | 6 | 0.270 | 0.0001 | 0.138 to 0.392 | Q=10.9; p=0.053; I^2^=54.2% |
| Speed of processing | 4 | 0.069 | 0.660 | -0.232 to 0.357 | Q=18.9; p=00001; I^2^=84.1% |
| Working memory | 3 | 0.228 | 0.023 | 0.032 to 0.407 | Q=5.1; p=0.076; I^2^=61.1% |

SSTICS: Subjective Scale to Investigate Cognition in Schizophrenia

**Supplementary Table 4: Sub-analyses on the scales used to assess depressive symptoms and their association with subjective complaints**

| Scale | Studies | Effect size (r) | P-value | 95% Confidence interval | Heterogeneity |
| --- | --- | --- | --- | --- | --- |
| CDSS | 6 | 0.254 | 0.012 | 0.057 to 0.433 | Q=34.9; p=0.0001; I^2^=85.7% |
| Other * | 2 | 0.543 | 0.0001 | 0.359 to 0.686 | Q=1.5; p=0.225; I^2^=32.1% |
| PANSS | 5 | 0.265 | 0.0001 | 0.176 to 0.350 | Q=1.1; p=0.897; I^2^=0.0% |

* One study used the Beck Depression Inventory, while the other used the Hospital Anxiety and Depression Scale

**Supplementary Table 5: Publication bias**

| ***Correlation with*** | Kendall’s tau | p (tau) | Egger’s test (t) | p (Egger) |
| --- | --- | --- | --- | --- |
| *Cognition* | -0.4 | 0.044 | 1.4 | 0.204 |
| *Positive symptoms* | -0.103 | 0.626 | 1.4 | 0.178 |
| *Negative symptoms* | -0.01 | 0.956 | 0.3 | 0.745 |
| *Depressive symptoms* | 0.256 | 0.222 | 1.7 | 0.122 |

**Supplementary Table 6: Factor Analyses of the SSTICS across studies**

| Authors | Sample | Number of Factors | Factor Labels | Type of Analysis |
| --- | --- | --- | --- | --- |
| Al Mugaddam, 2025 | 126 | 1 | Unidimensional model | Confirmatory Factor Analysis |
| Cella, 2020 * | 607 | 1 | Unidimensional model | Confirmatory Factor Analysis |
| Stratta, 2020 | 131 | 4 | Attention and Memory of Information | Exploratory Factor Analysis and Confirmatory Factor Analysis |
|  |  |  | Daily Living Cognition |  |
|  |  |  | Memory |  |
|  |  |  | Medical Memory |  |
| Stip, 2003 | 114 | 6 | Sustained executive functions | Exploratory Factor Analysis |
|  |  |  | Memory for information |  |
|  |  |  | Effort awareness |  |
|  |  |  | Daily life |  |
|  |  |  | Distractibility |  |
|  |  |  | Alertness (vigilance) |  |
| Potvin, 2017 | 82 | 5 | Memory | Exploratory Factor Analysis |
|  |  |  | Attention |  |
|  |  |  | Executive functions |  |
|  |  |  | Medical memory |  |
|  |  |  | Language and praxis |  |
| Johnson, 2009 | 105 | 6 | Distractibility | Exploratory Factor Analysis |
|  |  |  | Daily life |  |
|  |  |  | Semantic memory |  |
|  |  |  | Disorder consciousness |  |
|  |  |  | Working memory |  |
|  |  |  | Executive skills |  |
| Gopal, 2024 | 191 | 6 | Working memory and attention | Exploratory Factor Analysis |
|  |  |  | Memory for daily living |  |
|  |  |  | Explicit memory and language |  |
|  |  |  | Episodic memory and praxia |  |
|  |  |  | Memory and alertness |  |
|  |  |  | Semantic memory |  |
| Marucci, 2018 | 44 | 2 | Subjective Metacognition | Exploratory Factor Analysis |
|  |  |  | Objective Metacognition |  |

* This article used used the 14-item SSTICS-brief version

**Supplementary Figure 1: Publication bias regarding the potential correlation between the SSTICs and global cognitive performance**
